# The 5.8S pre‐rRNA maturation factor, M‐phase phosphoprotein 6, is a female fertility factor required for oocyte quality and meiosis

**DOI:** 10.1111/cpr.12769

**Published:** 2020-01-31

**Authors:** Rui‐Rui Peng, Li‐Li Wang, Wen‐Yi Gao, Feng‐Yu Zhu, Fan Hu, Wen‐Tao Zeng, Li‐Ya Shi, Xi‐Chen Chen, Jing‐Yang Cai, Dong Zhang, Zheng‐Rong Xia, Zhi‐Xia Yang

**Affiliations:** ^1^ Center for Reproductive Medicine Shandong Provincial Hospital Affiliated to Shandong University Jinan China; ^2^ State Key Lab of Reproductive Medicine Nanjing Medical University Nanjing China; ^3^ Animal Core Facility Nanjing Medical University Nanjing China; ^4^ The Second Affiliated Hospital Nanjing Medical University Nanjing China; ^5^ Analysis and Test Center Nanjing Medical University Nanjing China

**Keywords:** 5.8S Pre‐rRNA, female fertility factor, meiosis, M‐phase phosphoprotein 6, oocyte, quality

## Abstract

**Objectives:**

M‐phase phosphoprotein 6 (MPP6) is important for 5.8S pre‐rRNA maturation in somatic cells and was screened as a female fertility factor. However, whether MPP6 functions in oocyte meiosis and fertility is not yet known. We aimed to address this.

**Materials and Methods:**

Mouse oocytes with surrounded nucleus (SN) or non‐surrounded nucleus (NSN) were used for all experiments. Peptide nanoparticle‐mediated antibody transfection was used to deplete MPP6. Immunofluorescence staining, immunohistochemistry and live tracker staining were used to examine MPP6 localization and characterize phenotypes after control or MPP6 depletion. High‐fidelity PCR and fluorescence in situ hybridization (FISH) were used to examine the localization and level of 5.8S rRNAs. Western blot was used to examine the protein level. MPP6‐EGFP mRNA microinjection was used to do the rescue.

**Results:**

MPP6 was enriched within ovaries and oocytes. MPP6 depletion significantly impeded oocyte meiosis. MPP6 depletion increased 5.8S pre‐rRNA. The mRNA levels of MPP6 and 5.8S rRNA decreased within ageing oocytes, and MPP6 mRNA injection partially increased 5.8S rRNA maturation and improved oocyte quality.

**Conclusions:**

MPP6 is required for 5.8S rRNA maturation, meiosis and quality control in mouse oocytes, and MPP6 level might be a marker for oocyte quality.

## 1 INTRODUCTION

A high‐quality mature fully grown oocyte (FGO) is one prerequisite for a healthy newborn. Within the ovaries of mammals (such as mice), FGOs are arrested at the germinal vesicle (GV) stage and cannot maturate until LH surge comes. Luckily, FGOs can also maturate during in vitro maturation (IVM), providing a convenient and independent cellular model for the functional and mechanical study of so‐called female fertility factors.

Oocyte maturation is the process whereby the oocyte accomplishes meiosis (GV → GV breakdown (GVBD) → metaphase I (MI) → metaphase II (MII)). Meiosis is regulated by numerous fertility factors, among which maturation‐promoting factor (MPF, composed of cyclin B and cdk1),^1^ spindle checkpoint proteins^2^ and the anaphase‐promoting complex^3^ may be regarded as “master regulators.” MPF promotes GVBD and meiotic progression; spindle checkpoint proteins remain active to monitor attachment and tension on the kinetochores until all chromosomes align at spindle equators, and all homologous kinetochore pairs are bipolarly attached at metaphase; and the anaphase‐promoting complex ubiquitinizes and degrades cyclin B, cohesion and checkpoint proteins (Mads and Bubs) to promote the onset of anaphase.^1^, ^2^, ^3^, ^4^, ^5^ However, not all mature oocytes (MII) have normal subsequent events (ie fertilization and early embryo development), indicating that oocyte maturation requires additional fertility factors.

Based on recent knowledge, researchers agree that oocyte maturation should include cytoplasmic, nuclear and epigenetic maturation,^6^, ^7^, ^8^ and further studies are required to completely resolve the mechanism. Recently, researchers identified many potential female fertility factors through transcriptome^8^, ^9^, ^10^, ^11^ and proteome‐wide^12^, ^13^, ^14^ analyses. For loss‐of‐function studies, besides small interfering RNA knockdown, the protein‐depletion method using specific antibodies and trim21‐mediated protein degradation provided a powerful tool,^15^ which we have applied successfully to IVM oocytes.^16^, ^17^

Ribosomal RNAs (rRNAs), including 5S, 5.8S, 18S and 28S, are the main components of ribosomes in eukaryotes. rRNAs maintain the structure of ribosomes together with constitutive ribosomal proteins and also function as peptidyl transferases to catalyse the formation of peptide bonds between amino acids during protein translation. Mature rRNAs come from the multi‐step splicing of pre‐rRNAs. Specifically, from 5′ to 3′, the 45S pre‐rRNA contains 18S, 5.8S and 28S rRNA, and intermediate sequences between them. 45S pre‐rRNA is first spliced to separate out 18S rRNA, and then, the remaining part is spliced into 5.8S and 28S rRNAs. Many ribosome‐associated proteins participate in this processes.^18^ For example, in mitotic human somatic cells, poly(A)‐specific ribonuclease participates in 18S rRNA maturation. Knockdown of poly(A)‐specific ribonuclease or exogenous expression of a dead mutant (D28A) induces 18S pre‐rRNA accumulation in both the cytoplasm and nucleus.^19^ 5′‐3′ exonuclease Rrp17p binds to late pre‐60S ribosomes and is required for maturation of the 5′ ends of 5.8S and 25S rRNA.^20^ Ribosome synthesis factor Rrp5 participates in multiple steps of pre‐rRNA splicing; the C‐terminal domain is required for 18S rRNA synthesis, whereas the N‐terminal domain is required for 5.8S and 25S rRNA maturation.^21^ Studies of the involvement of specific proteins in rRNA maturation in meiotic oocytes are very scarce. Recently, it was shown that DDB1 and cullin‐4‐associated factor 13 (DCAF13) were rich in the nucleolus of non‐surrounded nucleolus (NSN) oocytes, but become undetectable in surrounded nucleolus (SN) oocytes. DCAF13 deletion inhibited nucleolus maturation through inhibiting protein synthesis without affecting mRNA transcription. The mechanism involved the participation of DCAF13 in 18S rRNA maturation.^22^

M‐phase phosphoprotein 6 (MPP6) was initially identified together with other MPPs by MPM2, a monoclonal antibody that recognizes a group of related M‐phase phosphorylation sites, including F‐phosphor‐P‐L‐Q.^23^, ^24^ These MPPs mostly had characteristic and distinct localization patterns during mitosis compared with the patterns during interphase. However, these MPPs do not share high sequence similarity or conserved domains, indicating their diversified functions.^23^, ^24^ Notably, MPP6 is the only MPP to be screened as a “female fertility factor” in *foxo3* ^‐/‐^ mouse ovaries.^25^ Several studies in somatic cells have shown that MPP6 plays an important role in the maturation of 5.8S rRNA, and the degradation of various other RNAs, by recruiting the nuclear exosome complex.^26^, ^27^, ^28^ However, there are no MPP6 studies in oocyte meiosis. In the present study, we found that MPP6 was enriched within ovaries and oocytes, and important for 5.8S rRNA maturation within oocytes. Furthermore, MPP6 was important for normal meiosis, fertilization and oocyte quality. Finally, decreased MPP6 and its mis‐localization appeared to be related to oocyte ageing.

## 2 MATERIALS AND METHODS

### 2.1 Chemicals and antibodies

All chemicals and reagents were obtained from Sigma unless otherwise stated.

Primary antibodies were mouse anti‐β‐actin (Cat#: A5316‐100); mouse anti‐GAPDH (Cat#: 30201ES60; YEASEN); mouse polyclonal a anti‐α‐Tubulin (cat#:G3115); mouse polyclonal a anti‐β‐Tubulin (cat#:sc‐5274); mouse monoclonal anti‐alpha tubulin (Acetyl Lys40) (cat#:bsm‐33235M; Bioss); rabbit polyclonal anti‐mphosh6 (cat#:10695‐1‐AP; Proteintech); rabbit polyclonal anti‐BubR1 (cat#:ab28193; Abcam); human anti‐centromere CREST (Cat#: 15‐234; Antibodies Incorporated); mouse polyclonal anti‐cyclin B1 (Cat#: B8566); rabbit polyclonal anti‐Phosphorylation CDK1 (Thr14) (Cat#: OAAN02724; Aviva Systems Biology); and rabbit anti‐Phosphorylation AKT (s473) (Cat#: 9271; Cell Signaling Technology). Dilution ratio for each primary antibody is in Table S1.

Fluorescent labelled secondary antibodies were all purchased from Jackson ImmunoResearch. The stock conc. was 1.5 mg/mL and used at 1:750 dilution: Cy2‐conjugated donkey anti‐mouse IgG (Code: 715‐225‐150); Cy2‐conjugated donkey anti‐rabbit IgG (Code: 711‐225‐152); Cy5‐conjugated donkey anti‐mouse IgG (Code: I715‐175‐150); and Rhodamine (TRITC)‐conjugated donkey anti‐rabbit IgG (Code: 711‐025‐152). Horseradish peroxidase (HRP)‐conjugated goat anti‐rabbit IgG and goat anti‐mouse IgG secondary antibodies were all purchased from Vazyme and used at 1:5000 dilution.

### 2.2 Oocyte collection and culture

GV oocytes were obtained from the ovaries of 3‐week‐old ICR mice supplied by the Animal Core Facility of Nanjing Medical University. The mice were euthanized by CO_2_ inhalation and cervical dislocation, and ovaries were isolated and placed in operation medium (Hepes) with 2.5 nM milrinone and 10% foetal bovine serum (FBS) (Gibco). Oocytes were removed from the ovary by puncturing the follicles with a hypodermic needle. Cumulus cells were washed off the cumulus‐oocyte complexes, and groups of 50 isolated denuded oocytes were placed in 100 μL droplets of culture medium under mineral oil in plastic dishes. The culture medium was MEM+ (MEM with 0.01 mM EDTA, 0.23 mM Na‐pyruvate, 0.2 mM penicillin/streptomycin, 3 mg/mL BSA) containing 20% FBS. Oocytes were grown at 37.0°C, 5% O_2_ and 5% CO_2_ in a humidified atmosphere. Before in vitro maturation (IVM), all culture media included 2.5 nM milrinone to prevent the resumption of meiosis. All experiments were approved by the Animal Care and Use Committee of Nanjing Medical University and were performed following institutional guidelines.

### 2.3 Antibody transfection

Chariot™ Protein Delivery Reagent (Active Motif) was used for antibody transfection. Briefly, two tubes, one containing 1 µl Chariot reagent (1 mg/mL in 50% DMSO) in 5 µl sterile water, and the other 1 µg antibody in PBS (final volume = 6 µl), were first set up. Next, both solutions were mixed together gently and incubated at room temperature for 30 min to allow the formation of Chariot‐IgG complexes, which were then added into the 100 µl MEM + droplet containing 50 oocytes. After 12‐14 h, the oocytes were washed to remove the complex‐containing MEM+, and after 1‐2 h, another two rounds of antibody transfection were repeated to ensure the effectiveness of antibody‐mediated inhibition. During the whole procedure, typically 40‐44 h long, 2.5 nM milrinone was always included to prevent the resumption of meiosis. Next, oocytes were transferred into milrinone‐free MEM + and cultured for 8 or 16 h, then subjected to the experiments described below. Antibodies for transfection were thoroughly buffer‐exchanged (over 10^4^ dilutions of original buffer) into PBS/50% glycerol with size‐exclusion spin columns (Millipore, cut‐off, 100 KDa; spin speed, 2400 *g*) to remove antiseptics (usually NaN_3_) contained in the original formulation.

### 2.4 In vitro Fertilization (IVF)

Spermatozoa were obtained from the epididymis of 10‐week‐old B6 x DBA2 F1 male mice and were then capacitated in 1ml MEM + for 1 hour. Control or MPP6‐DE oocytes were washed rapidly for 3 times with MEM + medium to remove FBS right before fertilization. Next, 10 µl of a sperm suspension containing 5‐10 × 10^6^/mL spermatozoa was added to 490 µl MEM + medium, and FBS‐free oocytes were added. 9 hours later, the oocytes were processed for immunofluorescence and examined to determine the frequency of successful fertilization, by the identification of the formation of pronuclei.

### 2.5 Mitochondrial staining and ATP measurements

For mitochondrial staining, oocytes were incubated in Hepes containing 100 nM Mito Tracker (Cat#: M7521, Invitrogen) and 10 µg/mL Hochest 33 342 (Sigma) for 30 min. Images were taken with an Andor Revolution spin disc confocal workstation.

For ATP measurements, the oocytes were first lysed with 100 µl ATP lysis solution (Cat#: S0026, Beyotime) on ice. The samples were then detected by enzyme‐labelled instrument Synergy2 (BioTek) to evaluate ATP level.

### 2.6 Detection of ROS generation

The ROS Assay Kit (Cat#: S0033, Beyotime) was used to detect ROS generation in oocytes. Briefly, oocytes were incubated with dichlorofluorescein diacetate (DCFH‐DA) probe for 20 min at 37°C in the dark, washed and mounted on slides for confocal imaging.

### 2.7 Apoptosis detection

Apoptosis was detected with an Annexin V‐FITC/PI Apoptosis Detection Kit (Cat#: 40302ES20, YEASEN). Oocytes were stained with 5 μL annexin V‐FITC and 10 μL propidium iodide (PI) staining solution diluted in 100 μL binding buffer for 15 min in the dark at RT. After washing, oocytes were mounted onto glass slides, and images were obtained as above.

### 2.8 Immunofluorescence staining

Oocytes were briefly washed in PBS with 0.05% polyvinylpyrrolidone (PVP), permeabilized in 0.5% Triton X‐100/PHEM (60 mM PIPES, 25 mM Hepes, pH 6.9, 10 mM EGTA, 8 mM MgSO4) for 5 min and washed three times rapidly in PBS/PVP. Next, the oocytes were fixed in 3.7% paraformaldehyde (PFA)/PHEM for 20 min, washed three times (10 min each) in PBS/PVP, and blocked with blocking buffer (1% BSA/PHEM with 100 mM glycine) at RT for 1 h. Oocytes were then incubated at 4°C overnight with primary antibody diluted in blocking buffer, washed three times (10 min each) in PBS with 0.05% Tween‐20 (PBST), incubated at room temperature for 45 min with secondary antibody diluted in blocking buffer (1:750 in all cases), and washed three times (10 min each) in PBST. Finally, DNA was stained with 3 µg/mL Hoechst 33 258, and the oocytes were mounted onto a slide with mounting medium (0.5% propyl gallate, 0.1M Tris‐HCl, pH 7.4, 88% glycerol) and covered with a cover glass (0.13‐0.17 µm thick). To maintain oocytes' structure, two strips of double‐stick tape (90 µm thick) were positioned between the slide and cover glass. Images were obtained as above.

### 2.9 Fluorescence in situ hybridization (FISH)

FISH was performed with the antisense oligonucleotide probes listed in Table S2. The probes were synthesized by Genewiz (Suzhou, Jiangsu, China) and labelled at the 5′ terminal end. The probes targeting ITS2 and 5'ETS of 5.8S pre‐rRNA were conjugated with FAM (green) and TAMRA (red), respectively. Oocytes were fixed with 70% ethanol for 30 min at room temperature and then transferred onto slides. The oocytes on the slides were quickly immobilized as 70% ethanol evaporated. Then, the slides were washed in saline‐sodium citrate buffer (2 × SSC, 0.3 M NaCl, 0.03 M Na_3_C_6_H_5_O_7_, pH 7.0) and incubated with hybridization mix solution. The hybridization mix contained 50% deionized formamide (Sigma‐Aldrich), 10% dextran sulfate (Loba Feinchemie GMBH), 5% 20 × SSC (3 M NaCl, 0.3 M Na_3_C_6_H_5_O_7_, pH 7.0), and 8 ng/μL probes. Hybridization was performed in a wet chamber for 18 h at 42°C. After hybridization, oocytes were sequentially washed with 50% formamide (Panreac) in 2 × SSC (3 × 10 min) at 42°C, 2 × SSC at 42°C (10 min), and 2 × SSC (10 min) at room temperature. The oocytes were counterstained with 1 μg/mL Hoechst 33 342 for 15 min, and then, mounting medium (same as in immunofluorescence staining) was added onto the oocytes and covered with a cover glass (0.13‐0.17 µm thick).

### 2.10 mRNA production and microinjection

For mRNA production, MPP6 and EGFP full‐length coding sequences were amplified and cloned into pBluescript II SK (+) at BamHI/EcoRV and EcoRV/XhoI restriction sites for a fusion expression of MPP6‐EGFP. The constructed plasmid was digested and linearized by PsiI, purified as a DNA template for MPP6‐EGFP mRNA transcription. T3 mMessage mMachine Ultra Kit (Ambion) was used to transcribe the initial MPP6‐EGFP mRNA, and then, Poly(A) Tailing Kit (Ambion) was used to elongate the 3′ UTR and stabilize the mRNA.

For microinjection, a thin‐wall glass tube with a built‐in guide wire (1.0 mm outer diameter, 0.8 mm inner diameter; World Precision Instruments) was pulled into two needles with Micropipette Puller P‐97 (Sutter Instrument). Then, the needle tip was bent on a Micro Forge (Narishige) at 30 degrees. MPP6‐EGFP mRNA (500‐1000 ng/µl) was loaded into the front part of the tip by guide wire‐assisted siphonage. Then, the needle was loaded onto the 3‐D electromechanical arm of a micromanipulator (Narishige) installed on a Ti‐S inverted fluorescence microscope (Nikon) and connected with a nitrogen‐driven programmable injector (Narishige). The injection time was about 10‐20 ms, and the injection volume was about 10‐20 pl.

### 2.11 Data analysis and statistics

All experiments were repeated at least three times. Measurements of immunohistochemistry images, immunofluorescence images, FISH images, Western blot and PCR bands were all performed with ImageJ (NIH). Net intensity was obtained by subtracting mean object intensity by mean background intensity around the object. Data were presented as mean ± SEM. Statistical comparisons between two groups were made with Student's t test. Statistical significance was set at *P* < .05.

## 3 RESULTS

### 3.1 The female fertility factor MPP6 is important for 5.8S rRNA maturation in FGOs

**Figure 1:**
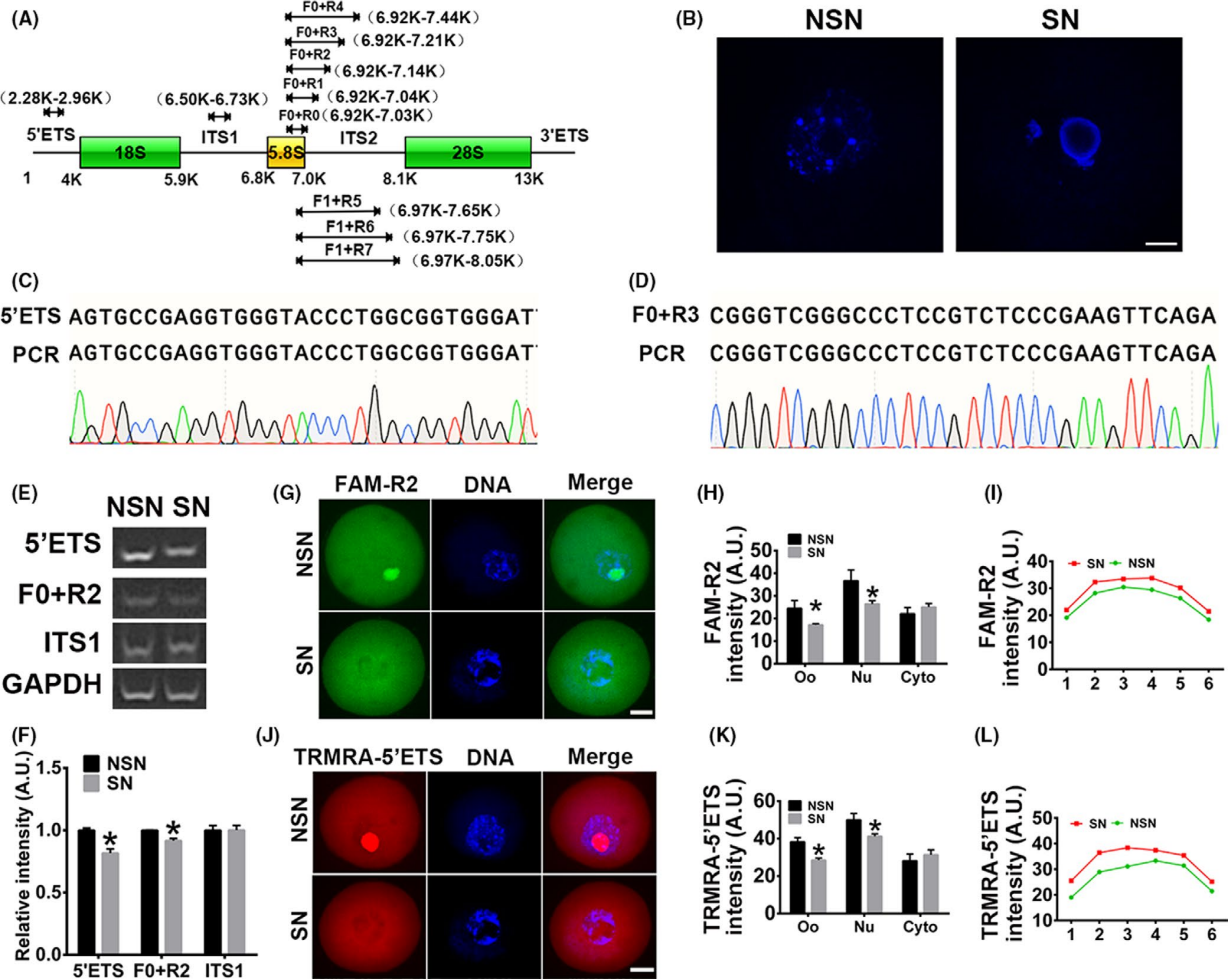
MPP6 is important for 5.8S rRNA processing. (A) Multiple primer pairs were designed to amplify different regions of pre‐rRNA (Detailed information about sequences and target regions is in Table S2). (B) FGOs (fully grown oocytes) from large antral follicles were stained with live‐cell dye hoechst 33 258 and then strictly subtyped into NSN (non‐surrounded nucleus) FGOs and SN (surrounded nucleus) FGOs for subsequent experiments. (C and D) Sanger sequencing showed that PCR product with 5'ETS (C) or F0 + R3 (D) primer in the SN FGOs was identical to the 5'ETS pre‐rRNA or 5.8S pre‐rRNA. (E) RT‐PCR showed that strong bands can be detected by any of primer pairs above (A) in both NSN and SN, but only products with 5'ETS primer and F0 + R2, not with ITS1, significantly decreased in SN FGOs. GAPDH was the loading control. (F) Quantification of E. (G) Fluorescence in situ hybridization (FISH) of pre‐5.8S rRNA with FAM‐R2 probe showed that in NSN oocytes, 5.8S pre‐rRNA was enriched within nucleus; while in SN oocytes, 5.8S pre‐rRNA significantly decreased within nucleus but tended to increased within the cytoplasm around nucleus. (H) Quantification of FAM‐R2 intensity of oocytes (Oo), nucleus (Nu) and cytoplasm (Cyto). (I) Representative intensity curve of cytoplasmic FAM‐R2 showed that FAM‐R2 intensity was higher around the nucleus in SN oocytes than in NSN oocytes. (J) FISH of pre‐rRNA with TRMRA‐5'ETS probe showed that in NSN oocytes, 5'ETS pre‐rRNA was enriched within nucleus; while in SN oocytes, 5'ETS pre‐rRNA significantly decreased within nucleus but tended to increased within the cytoplasm around nucleus. (K) Quantification of TRMRA‐5'ETS intensity of oocytes (Oo), nucleus (Nu) and cytoplasm (Cyto). (L) Representative intensity curve of cytoplasmic TRMRA‐5'ETS showed that TRMRA‐5'ETS intensity was higher around the nucleus in SN oocytes than in NSN oocytes. Scale bar, 20 µm. *P < .05

Nuclear exosome complex is required for the maturation of 5.8S rRNA in human somatic cells, while MPP6 is a nucleolus‐specific exosome co‐factor that binds preferentially to poly(C) and (U)‐rich ITS2 region to recruit the exosome.^26^, ^27^, ^28^ For SN FGOs that are transcriptionally quiescent, some researches suggested that rDNA‐containing nucleolus‐like bodies did not contain transcribed rRNA genes, pre‐rRNAs or pre‐ribosomes.^29^, ^30^, ^31^ Thus, we first wanted to verify whether pre‐rRNA existed within SN FGOs. Due to the low homology of the ITS2 region between human and mouse (Figure S1), we could not deduce which subsection of the ITS2 region MPP6 might bind in the mouse. Thus, we designed two forward primers (F0 and F1) inside the mature 5.8S rRNA, and eight reverse primers within mature 5.8S rRNA (R0) or different poly(C) or (U)‐rich ITS2 regions of pre‐rRNA (R1‐R7, Figure 1A, Table S2). We also designed a pair of primers for the 5'ETS and ITS1 regions of pre‐rRNA.

We first strictly subtyped FGOs into NSN and SN FGOs (Figure 1B). Real‐time PCR showed that the 5.8S pre‐rRNA amplified by any of the primer pairs existed in both types of FGOs. Sanger sequencing of the PCR products with 5'ETS and 5.8S F0 + R3 primer pairs confirmed that the products were identical to the 5'ETS and 5.8S rRNA + ITS2 regions (Figure 1C,D). The levels of pre‐rRNAs amplified with 5'ETS and 5.8S F + R2 were significantly higher in NSN than SN FGOs (Figure 1E,F, 5'ETS intensity, NSN vs SN, 1.00 vs 0.81; F0 + R2 intensity, 1.00 vs 0.91), suggesting that the R2 poly (C) and (U) region might be where MPP6 binds to start its work.

The PCR results only reflected the gross rRNA quantity, including nuclear and cytoplasmic rRNAs. To verify the presence and dynamic changes of 5.8S rRNA more precisely, we synthesized two DNA probes with fluorescent dyes FAM and TRMRA label at the 5' terminus, covering the R2 sequence and the reverse primer of 5'ETS, respectively. Then, we performed rRNA fluorescence in situ hybridization (FISH) and found that the gross 5.8S rRNA intensity detected by FAM‐R2 and TRMRA‐5'ETS were significantly higher in the NSN oocytes than in the SN oocytes (FAM‐R2 oocyte intensity, Figure 1G,H, NSN vs SN, 24.49 vs 17.17; TRMRA‐5'ETS intensity, Figure 1J,K, NSN vs SN, 38.26 vs 28.54). Next, we measured nuclear and cytoplasmic rRNA intensity separatively. We found that nuclear rRNA intensity was significantly higher in the NSN oocytes than in the SN oocytes for both probes (FAM‐R2 nuclear intensity, Figure 1G,H, NSN vs SN, 36.66 vs 26.44; TRMRA‐5'ETS intensity, Figure 1J,K, NSN vs SN, 50.01 vs 41.25). The cytoplasmic rRNA intensity, although not significantly, tended to be higher in SN than in NSN oocytes (Figure 1G,H,J,K). Representative plots of rRNA intensity in 6 cytoplasmic subregions more intuitively showed that the cytoplasmic FAM‐R2 and TRMRA‐5'ETS intensity around the nucleus tended to increase at a larger scale than the intensity near the cortex in SN oocytes (Figure 1I,L). These results indicated that pre‐rRNA existed in both SN and NSN of FGOs, and the distribution of pre‐rRNA was very dynamic as FGOs transited from NSN to SN.

Next, we examined the level and distribution of MPP6. The relative MPP6 mRNA level in NSN FGOs was significantly higher than in SN FGOs (Figure S2A,B, MPP6 mRNA intensity, NSN vs SN, 1.00 vs 0.79). Although Western blots showed no difference for the total MPP6 protein level between NSN and SN oocytes (Figure S2C,D), immunofluorescence showed that MPP6 intensity in NSN oocytes was significantly higher than in SN oocytes (Figure S2E,F, oocyte MPP6 intensity, NSN vs SN, 43.12 vs 36.12). And separative measurements of nuclear and cytoplasmic MPP6 intensity showed that the nuclear MPP6 in NSN oocytes was significantly higher than in SN oocytes, while cytoplasmic MPP6 intensity tended to be higher in SN oocytes than in NSN oocytes (Figure S2E,F, nuclear MPP6 intensity, NSN vs SN, 59.05 vs 40.42). These results indicated that the distribution of MPP6, similar to pre‐rRNAs, was very dynamic as FGOs transited from NSN to SN.

### 3.2 MPP6 is rich in oocytes and important for their in vitro maturation

**Figure 2:**
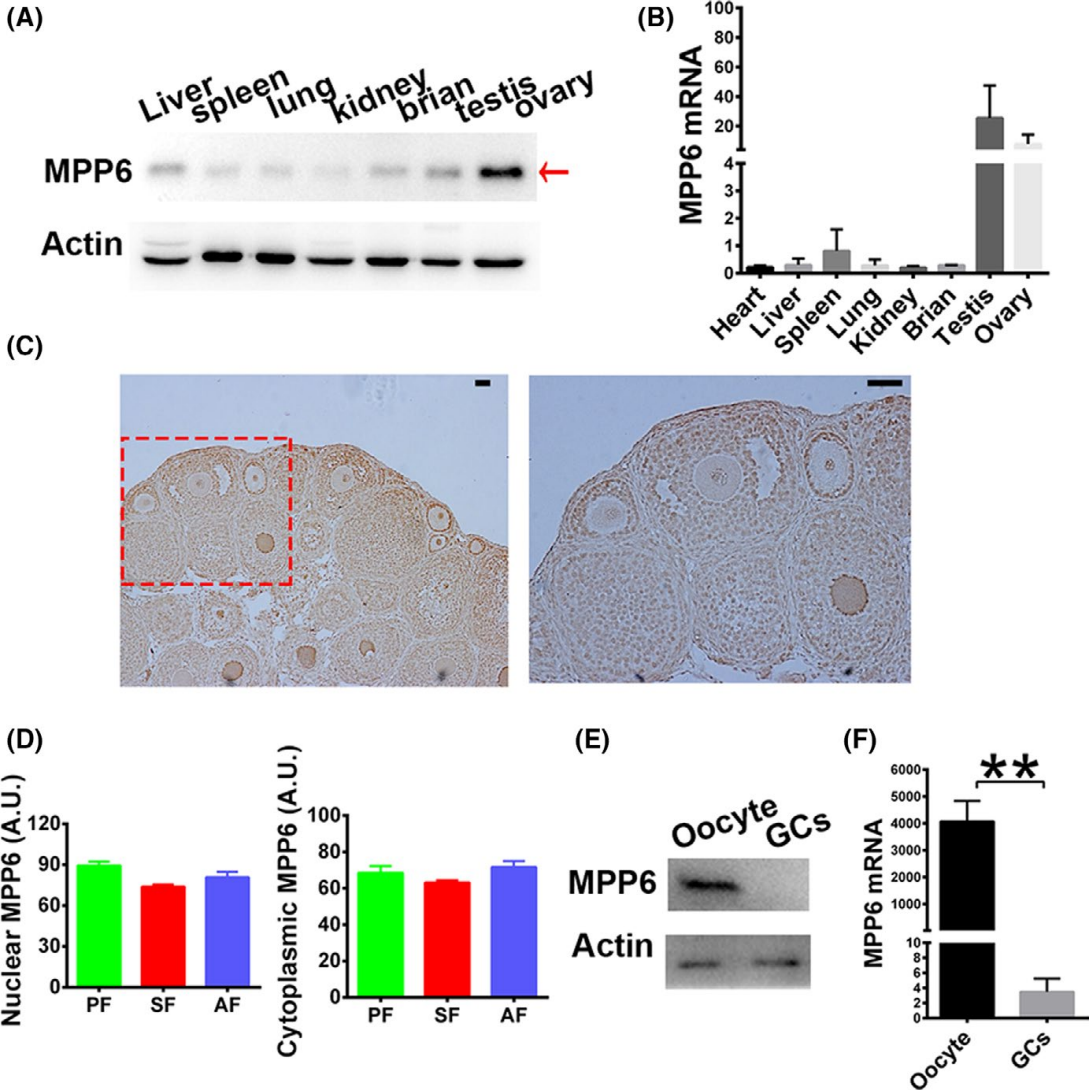
MPP6 enriches within ovaries and oocytes. (A) Western blot showed that MPP6 was rich within ovaries. (B) Q‐PCR showed that MPP6 was rich within ovaries. (C) Immunohistochemistry showed that MPP6 level kept constant as follicles develop from primordial to antral stage. (D) Quantification of (C). PF, primordial follicle; SF, secondary follicle; AF, antral follicle. (E) Western blot showed that MPP6 protein is more abundant in oocytes than in granulosa cells (GCs). (F) Q‐PCR showed that MPP6 mRNA is more abundant in oocytes than in granulosa cells. Scale bar, 50 µm. **P < .01

MPP6 was screened as a female fertility factor and found to be abundant in ovaries and oocytes in a transcriptome study.^25^ However, there are no studies to verify this finding. We found that MPP6 was abundant within ovaries at both the mRNA and protein levels (Figure 2A,B). Immunohistochemistry also showed that MPP6 was enriched in oocytes (Figure 2C), and the abundance remained constant as the follicles developed (Figure 2C,D). Furthermore, MPP6 was more abundant within oocytes than in granulosa cells (Figure 2E,F). These findings suggested that MPP6 might be important for oocyte maturation.

**Figure 3:**
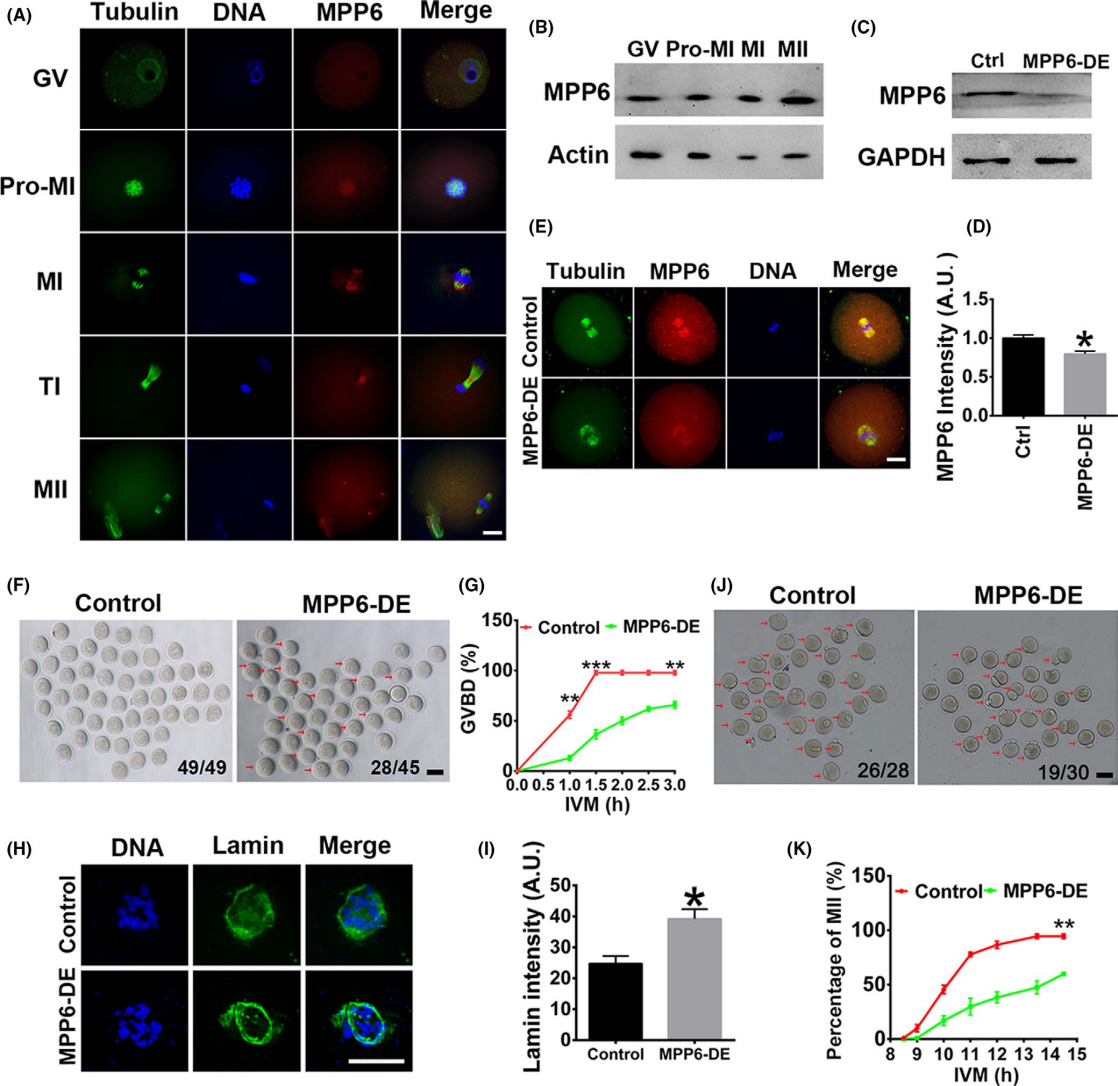
MPP6 depletion impeded meiotic progression. (A) Immunofluorescence showed that MPP6 enriched within the nucleus at the GV stage, within spindles at GVBD and MI stage, at cytokinesis ring at the TI stage, and at spindle poles at MII stage. DNA in blue, tubulin in green, MPP6 in red. (B) Western blot showed that the MPP6 protein level remained constant during meiosis. (C) Western blot showed that chariot‐mediated antibody transfection significantly reduced the endogenous MPP6 level. (D) Quantification of (C). (E) Immunofluorescence showed that MPP6 intensity was significantly reduced by chariot‐mediated antibody transfection. Tubulin in green, MPP6 in red. (F) Bright‐field (BF) image showed that MPP6 depletion significantly decreased the percentage of GVBD oocytes at 3 hours of in vitro maturation (IVM). Numbers in the images indicate the number of GVBD oocytes/number of total oocytes. (G) Quantification of GVBD rate at different IVM time points (0‐3 hours). (H) Immunofluorescence showed that at 2.5 hours of IVM, when GVBD was about to occur, MPP6 depletion significantly increased lamin intensity on the nuclear envelope. DNA in blue, lamin in green. (I) Quantification of (H). (J) Bright‐field (BF) image showed that MPP6 depletion significantly decreased the percentage of MII oocytes at 14.5 hours of IVM. Numbers in the images indicate the number of MII oocytes/number of total oocytes. (K) Quantification of MII rate (1PB) at different IVM time points (8‐14.5 hours). Scale bars in A, E and H, 20 µm. Scale bar in F and J, 100 µm. *P < .05; **P < .01; ***P < .001

Immunofluorescence showed that MPP6 was enriched within nuclei at the GV stage, and within spindles at the MI and MII stages. The abundance remained constant during meiosis, indicating that it might be involved in this process (Figure 3A,B).

We used chariot‐mediated antibody transfection to effectively reduce the endogenous MPP6 level (Figure 3C‐E) and examined the in vitro maturation of oocytes. MPP6 depletion (MPP6‐DE) significantly reduced the percentage of GVBD (Figure 3F,G, the percentage of GVBD at 3h, Ctrl vs MPP6‐DE, 0.97 vs 0.65). Increased lamin is known to stabilize the nuclear envelope.^32^ In further support of the GVBD results, we examined lamin distribution at 2.5 h of IVM, when control oocytes were about to undergo GVBD while the nuclear envelope was still present. Results showed that the envelope lamin intensity in MPP6‐depleted oocytes was significantly higher than in control oocytes (Figure 3H,I, lamin intensity at 2.5 h, Ctrl vs MPP6‐DE, 24.74 vs 39.20). And MPP6 depletion also significantly decreased the percentage of MII (Figure 3J,K, the percentage of MII at 14.5 h, Ctrl vs MPP6‐DE, 0.94 vs 0.59). These results indicated that MPP6 was predominant in oocytes and necessary for oocytes to enter meiosis.

### 3.3 MPP6 depletion causes abnormal spindle organization, chromosome compression, checkpoint activation and fertilization

**Figure 4:**
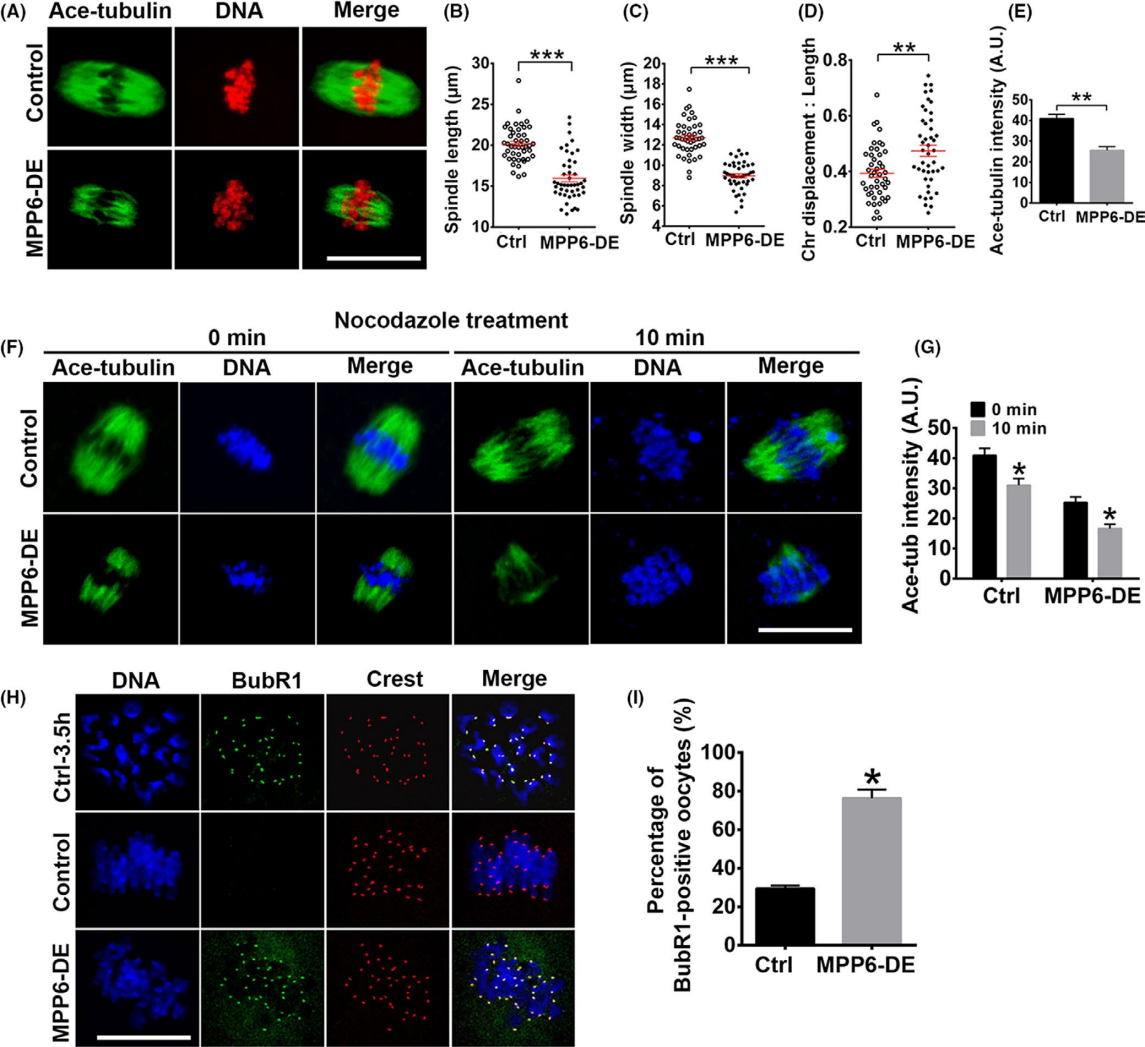
MPP6 depletion caused spindle disorganization and spindle checkpoint activation. (A) Immunofluorescence showed that MPP6 depletion significantly altered the spindle geometry, decreased spindle microtubule intensity and increased the chromosome displacement at 7.5 hour of IVM. Tubulin (green) was detected by the acetylated tubulin antibody. (B‐D) Quantification of A. MPP6 depletion significantly decreased spindle length and width and increased the ratio of chromosome displacement/spindle length. (E) Quantification showed that MPP6 depletion significantly reduced acetylated tubulin intensity. (F) The speed of microtubule disassembly by nocodazole in MPP6‐depleted oocytes was significantly faster than in control oocytes. Oocytes were stained with acetylated tubulin antibody. (G) Quantification of (F). (H) BUBR1 staining on kinetochores in MI oocytes significantly increased after MPP6 depletion. DNA in blue, BUBR1 in green, kinetochores in red. (I) Quantification of (H) the percentage of BUBR1‐positive oocytes significantly increased. Scale bar, 20 µm. *P < .05; **P < .01; ***P < .001

Because MPP6 depletion significantly disturbed the progression of meiosis, we next examined how it functioned. At 7.5 h of IVM, most FGOs in the control group had progressed into the metaphase I (MI) stage, and all chromosomes were well aligned at the equatorial plate. MPP6 depletion significantly decreased spindle length (Figure 4A,B, spindle length, Ctrl vs MPP6‐DE, 19.75 µm vs 15.10 µm) and width (Figure 4A,C, spindle width, Ctrl vs MPP6‐DE, 12.76 µm vs 8.95 µm), and increased chromosome displacement (Figure 4A,D, chromosome displacement/spindle length, Ctrl vs MPP6‐DE, 0.39 vs 0.47). These results suggested that spindle microtubules might be less stable.

Acetylation is a major modification of tubulin within oocyte spindles. Thus, we also measured the acetylated tubulin (ace‐tubulin) level. MPP6 depletion significantly reduced ace‐tubulin intensity (Figure 4A,E, ace‐tubulin intensity, Ctrl vs MPP6‐DE, 41.01 vs 25.66). Less stable microtubules are more susceptible to the microtubule‐depolymerizing drug, nocodazole. So we treated oocytes with this drug for 10 min. Results showed that the decreasing speed of ace‐tubulin intensity in MPP6‐depleted oocytes (Figure 4F,G, ace‐tubulin intensity in MPP6‐DE group, 0 min vs 10min, 25.32 vs 16.63, a 34.32% decrease) was significantly faster than in control oocytes (Figure 4F,G, ace‐tubulin intensity in Ctrl group, 0 min vs 10 min, 40.95 vs 30.99, a 24.32% decrease). Moreover, at MI, each homologous kinetochore pair was bi‐laterally attached by kinetochore microtubules. Thus, spindle checkpoint proteins, such as BubR1 and MAD2, will be inactivated and released from the kinetochores.^2^ MPP6 depletion also significantly increased the percentage of oocytes with strong BuBR1 signals at chromosomes by over 1.5‐fold (Figure 4H,I, percentage of BubR1‐positive oocytes, Ctrl vs MPP6‐DE, 29.51% vs 76.43%). Together, these results indicated that MPP6 depletion resulted in less stable microtubules and thereby more disorganized chromosomes.

**Figure 5:**
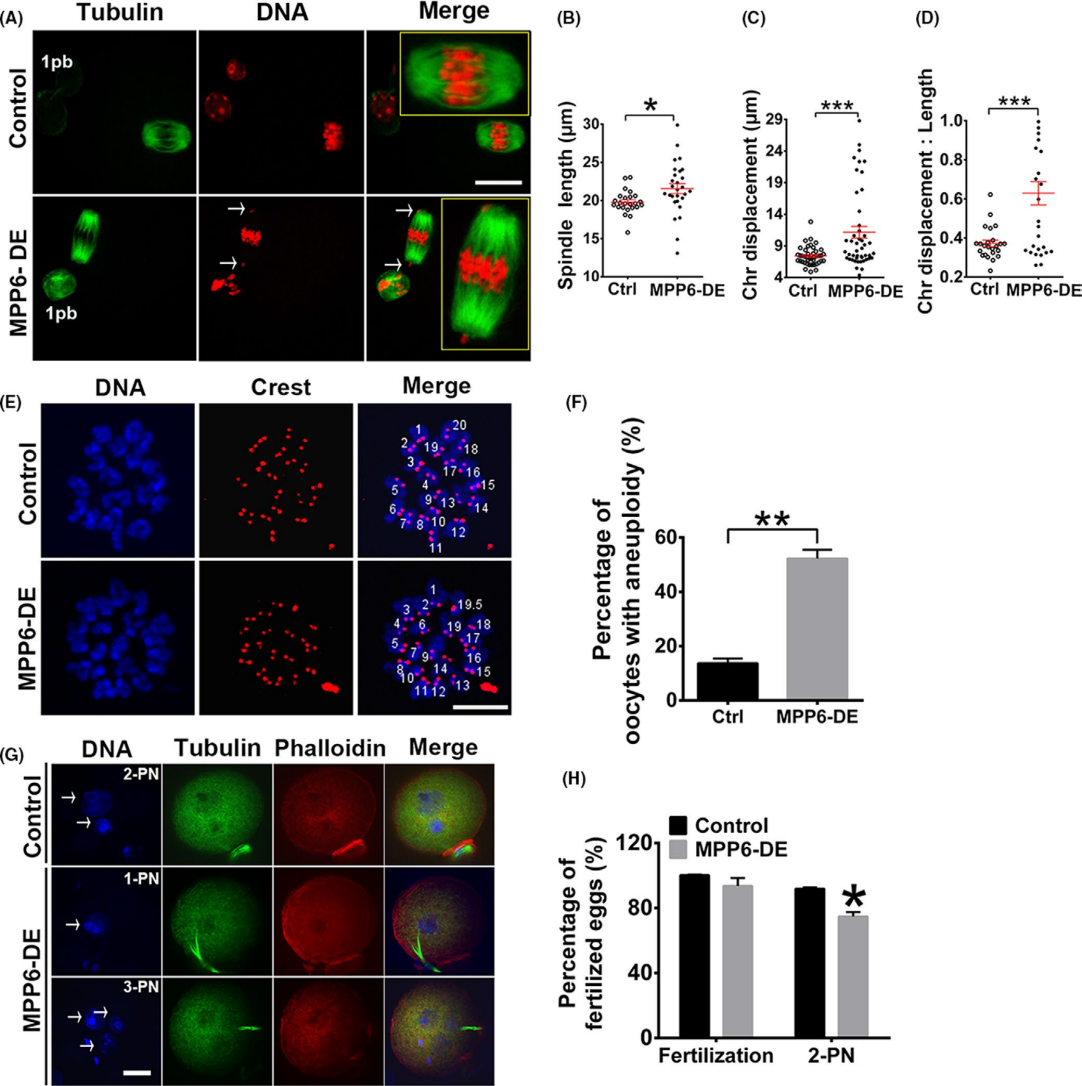
MPP6 depletion caused abnormality in oocyte maturation and fertilization. (A) At 14.5 hours of IVM, MPP6 depletion significantly altered the MII spindle geometry and disrupted chromosome congression. Tubulin in green, DNA in red. Arrows marked uncompressed chromosomes. (B‐D) Quantification of A. MPP6 depletion significantly increased spindle length, chromosome displacement and the ratio of chromosome displacement/spindle length. (E) Chromosome spreading of MII oocytes and kinetochore counting showed that the percentage of MII oocytes with aneuploidy significantly increased. DNA in blue, kinetochores in red. (F) Quantification of (E). (G) Percentage of fertilized oocytes with 2‐PN (2 pronuclear) significantly reduced after MPP6 depletion. DNA in blue, tubulin in green, F‐actin (stained with phalloidin) in red. Arrows marked pronucleus. (H) Quantification of (G). Scale bar, 20 µm. *P < .05; **P < .01; ***P < .001

At 14.5 h of IVM, most FGOs in the control group had progressed into the MII stage, and again, chromosomes were well‐compressed at the equatorial plates. Immunofluorescence showed that MPP6 depletion significantly altered the MII spindle geometry including length (Figure 5A,B, spindle length, Ctrl vs MPP6‐DE, 19.50 µm vs 21.55 µm), chromosome displacement (Figure 5A,C, chromosome displacement, Ctrl vs MPP6‐DE, 7.21 vs 10.07) and the chromosome displacement/spindle length ratio (Figure 5A,D, chromosome displacement/spindle length, Ctrl vs MPP6‐DE, 0.35 vs 0.62). Consequently, MPP6 depletion significantly increased the percentage of oocytes with aneuploidy (Figure 5E,F, percentage of oocytes with aneuploidy, Ctrl vs MPP6‐DE, 13.75% vs 52.27%) and decreased the percentage of normal oocyte fertilization (Figure 5G,H, percentage of fertilized oocytes with 2 pronuclear, Ctrl vs MPP6‐DE, 91.93% vs 74.83%), presumably due to these scattered chromosomes. Overall, these results suggested that MPP6 is important for spindle integrity, chromosome compression and checkpoint inactivation during meiotic metaphase.

### 3.4 MPP6 is important for oocyte quality in multiple ways

The meiotic spindle is not only the important apparatus for chromosome segregation, but also an important cytoskeletal structure for various cellular processes, including activation and inactivation of diverse microtubule‐associated kinases. Thus, presumably, spindle disruption caused by MPP6 depletion could affect various important cellular processes and meiotic kinases, and consequently affect oocyte quality.

**Figure 6:**
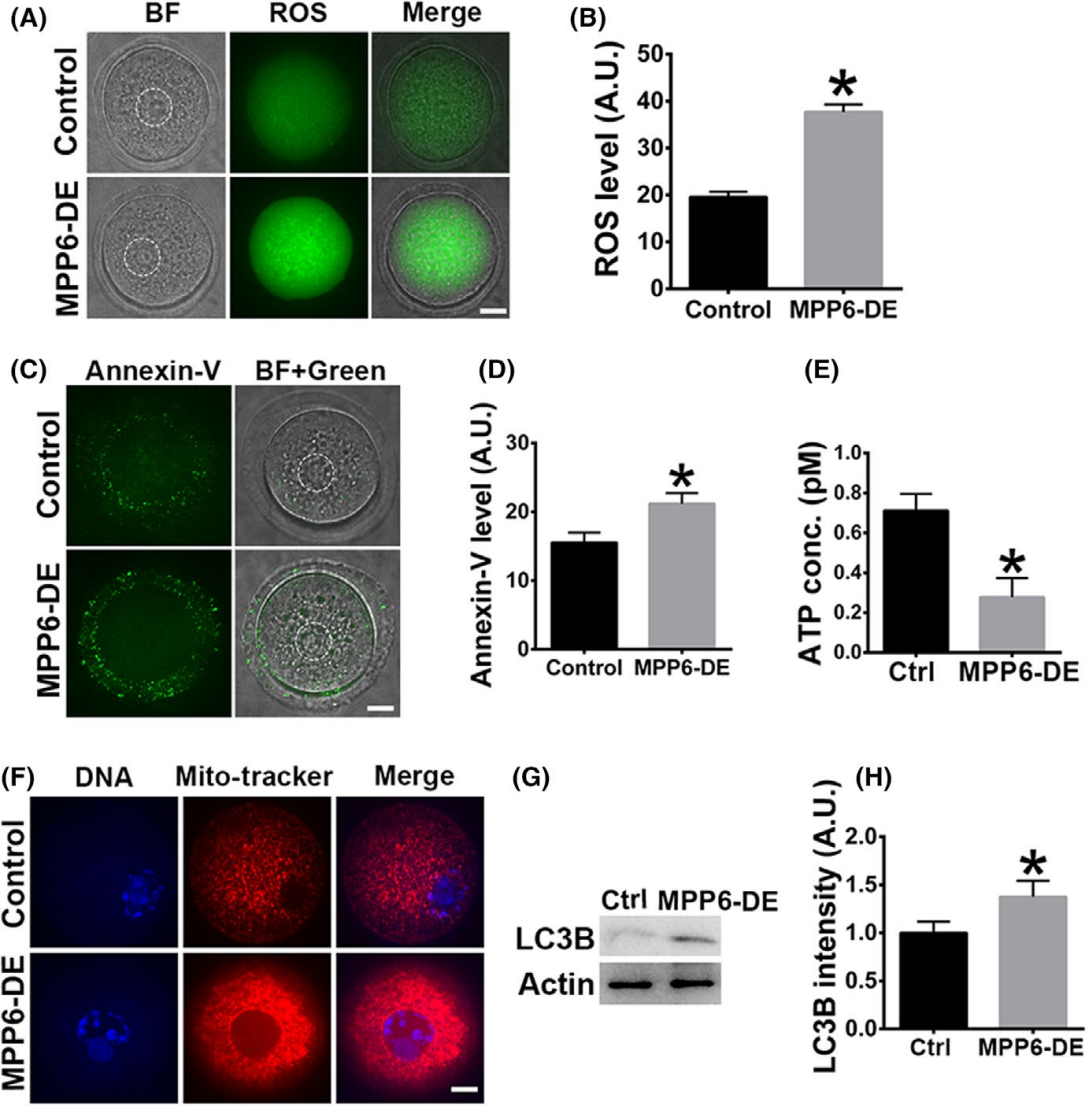
MPP6 is important for the normal levels of ROS, apoptosis, mitochondria and ATP. (A) MPP6 depletion significantly increased ROS level. The dot‐line circles marked the germinal vesicle (GV). (B) Quantification of (A). (C) MPP6 depletion significantly increased annexin V level. The dot‐line circles marked the germinal vesicle (GV). (D) Quantification of (C). (E) ATP level significantly decreased after MPP6 depletion. (F) Mitochondria intensity within the MPP6‐depleted oocyte significantly increased, and mitochondria formed big aggregates after MPP6 depletion. DNA in blue, mitochondria in red. (G) LC3B protein level significantly increased after MPP6 depletion. (H) Quantification of (G). Scale bar, 20 µm. *P < .05

To assess these possibilities, we examined the levels of reactive oxygen species (ROS), annexin V, and ATP, and the distribution of mitochondria, indices that are closely correlated with overall oocyte quality. ROS are toxic by‐products of aerobic metabolism, an increased level of ROS indicates abnormal aerobic metabolism.^33^ Annexin V is a marker of early apoptosis, and an increased level of annexin V indicates an abnormally high apoptotic level.^34^ Mitochondria are the factory that generates ATP, over‐aggregated mitochondria indicate impeded mitochondrial autophagy, and the cellular ATP level might decrease consequently.^35^ Our results showed that MPP6 depletion significantly elevated the ROS (Figure 6A,B, ROS level, Ctrl vs MPP6‐DE, 19.59 vs 37.68) and annexin V (Figure 6C,D, annexin V intensity, Ctrl vs MPP6‐DE, 15.53 vs 21.21) levels. The density of mitochondria within MPP6‐depleted oocytes significantly increased, and mitochondria formed large aggregates (Figure 6E). Consequently, the ATP level decreased over 1.5‐fold (Figure 6F, ATP conc., Ctrl vs MPP6‐DE, 0.71 pM vs 0.27 pM). Finally, Western blot showed that MPP6 depletion significantly increased LC3B level (Figure 6G,H, LC3B intensity, Ctrl vs MPP6‐DE, 1.00 vs 1.37), indicating that the autophagy was impeded.

**Figure 7:**
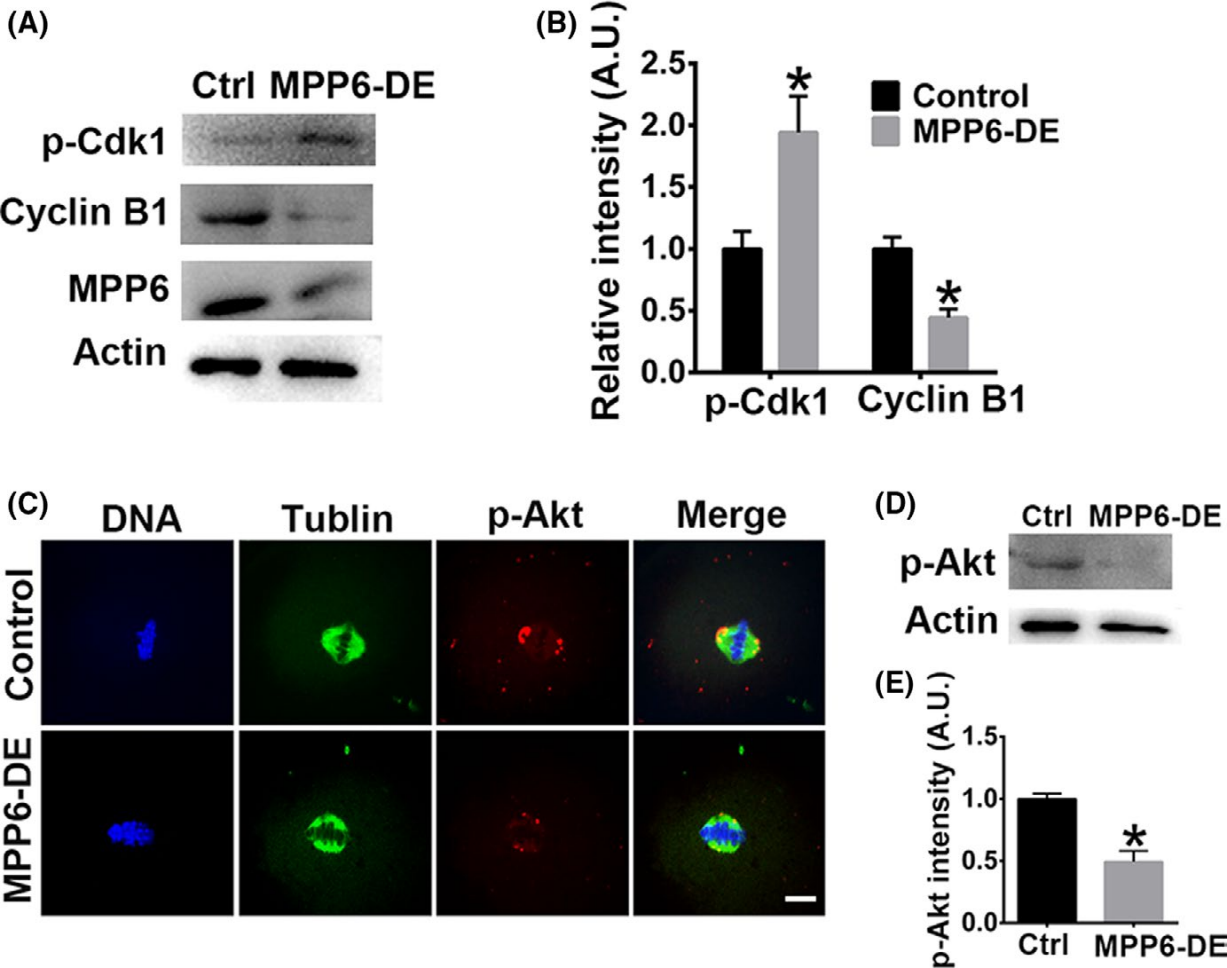
MPP6 is important for the normal activities of key meiotic kinases. (A) MPP6 depletion significantly increased p‐cdk1 level and decreased cyclin B1 level. (B) Quantification of (A). (C) Immunofluorescence staining showed that MPP6 depletion significantly reduced p‐Akt at spindle poles. DNA in blue, tubulin in green, p‐Akt in red. (D) Western blot showed that MPP6 depletion significantly reduced p‐Akt level. (E) Quantification of (D). Scale bar, 20 µm. *P < .05

Next, we examined the activities of key meiotic kinases. MPF, composed of p‐cdk1 and cyclin B1, is the master promoter of meiosis. Low MPF activity caused by increased p‐cdk1 or decreased cyclin B1 could retard meiosis.^36^, ^37^ Akt is an important meiotic kinase that regulates the progression of meiosis in FGOs.^38^, ^39^ Retarded or disrupted meiosis could subsequently affect many aspects of oocyte quality. Western blot showed that MPP6 depletion significantly increased p‐cdk1 by almost 100% (Figure 7A,B, p‐Cdk1 intensity, Ctrl vs MPP6‐DE, 1.00 vs 1.94) and decreased cyclin B1 by more than 50% (Figure 7A,B, Cyclin B1 intensity, Ctrl vs MPP6‐DE, 1.00 vs 0.44), indicating that MPF activity was significantly reduced. MPP6 depletion also significantly reduced p‐Akt by 50% (Figure 7C‐E, p‐Akt intensity, Ctrl vs MPP6‐DE, 1.00 vs 0.49). Overall, these results indicate that MPP6 is important for oocyte quality in various ways.

### 3.5 MPP6 is important for 5.8S rRNA maturation

**Figure 8:**
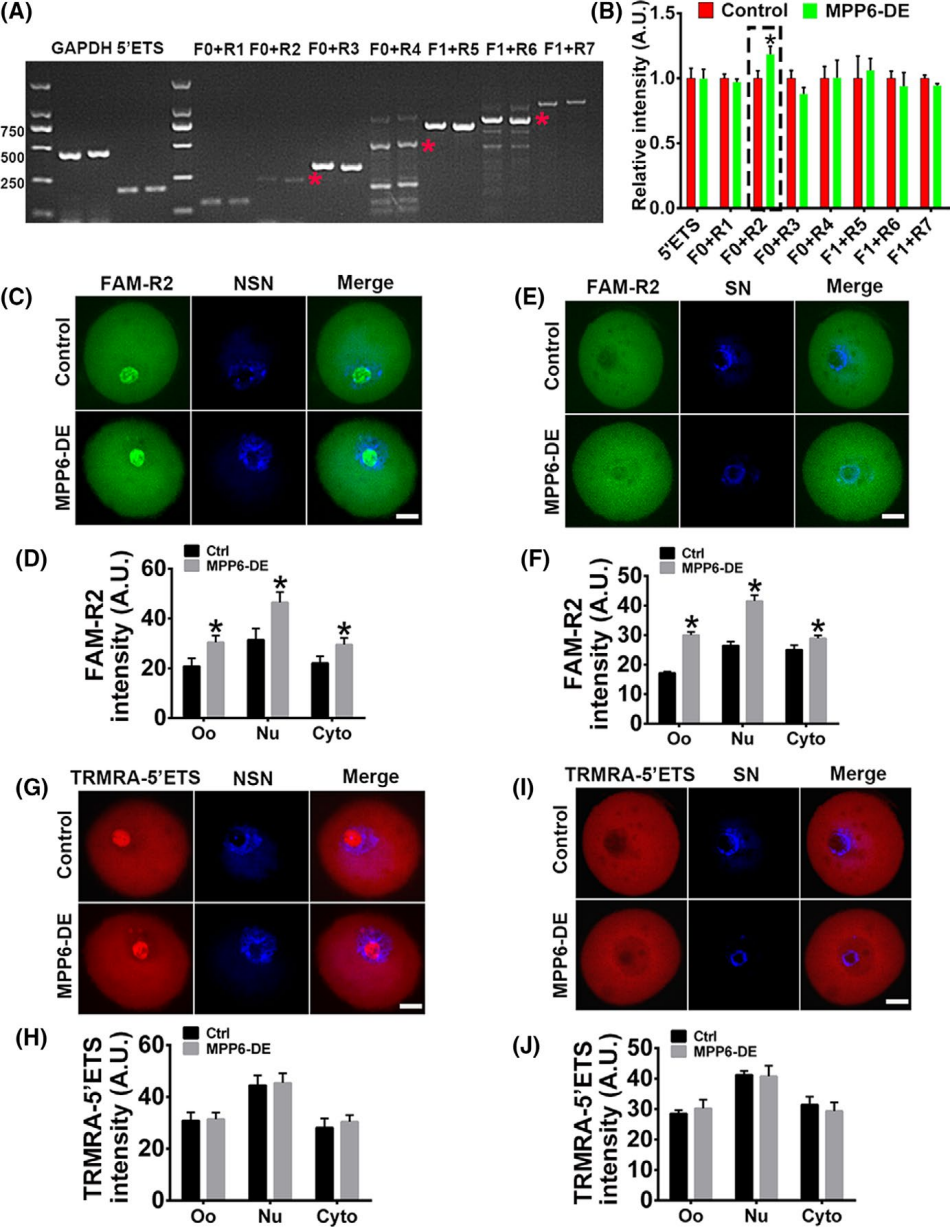
MPP6 is important for 5.8S rRNA maturation. (A) RT‐PCR showed that MPP6 depletion specifically increased 5.8S pre‐rRNA amplified with F0 + R2 primer pair. (B) Quantification of (A). (C) FISH of pre‐rRNA with FAM‐R2 probe in NSN oocytes showed that MPP6 depletion caused a significant increase of 5.8S pre‐rRNA within oocytes, nucleus and cytoplasm. (D) Quantification of (C). (E) FISH of pre‐rRNA with FAM‐R2 probe in SN oocytes showed that MPP6 depletion caused a significant increase of 5.8S pre‐rRNA within oocytes, nucleus and cytoplasm. (F) Quantification of (E). (G) FISH of pre‐rRNA with TRMRA‐5'ETS probe in NSN oocytes showed that MPP6 depletion did not cause a significant increase of 5'ETS pre‐rRNA within oocytes, nucleus and cytoplasm. (H) Quantification of (G). (I) FISH of pre‐rRNA with TRMRA‐5'ETS probe in SN oocytes showed that MPP6 depletion did not cause a significant increase of 5'ETS pre‐rRNA within oocytes, nucleus and cytoplasm. (J) Quantification of I. Scale bar, 20 µm. *P < .05

From the above evidence, and previous reports in somatic cells, it appears that MPP6 is related to 5.8S rRNA processing in oocytes. Next, we examined whether MPP6 depletion affected 5.8S rRNA maturation and which region it affected. RT‐PCR showed that MPP6 depletion specifically and significantly increased 5.8S pre‐rRNA amplified with the F + R2 primer (Figure 8A,B). For further verification, we performed rRNA FISH with the FAM‐R2 (covering the R2 primer) and TRAMA‐5'ETS probes. The results showed that MPP6 depletion significantly increased gross 5.8S rRNA intensity with the FAM‐R2 probe in both NSN oocytes (Figure 8C,D) and SN oocytes (Figure 8E,F). Particularly, FAM‐R2 intensity in MPP6‐depleted oocytes increased synchronously within nucleus and cytoplasm (Figure 8C‐F). In contrast, MPP6 depletion had no significant effect on pre‐rRNA probed with TRAMA‐5'ETS (Figure 8G‐J). These results indicated that MPP6 specifically promoted 5.8S rRNA maturation, and the binding site might be near primer R2.

### 3.6 The ageing‐associated decrease in oocyte quality partially correlates with a reduced MPP6 level

Numerous clinical studies have shown that the fertility of women starts to decrease at the age of 35, and, concomitantly, the oocyte quality also starts to decline.^40^, ^41^, ^42^, ^43^ A transcriptome study showed that 40 of 78 downregulated genes in the ovaries of ageing mice were ribosome‐related genes,^43^ indicating that downregulation of ribosome function might have a significant impact on fertility. Thus, we next investigated whether ageing could alter 5.8S rRNA maturation and whether the level of MPP6, the 5.8S pre‐rRNA maturation factor, could be affected by ageing.

**Figure 9:**
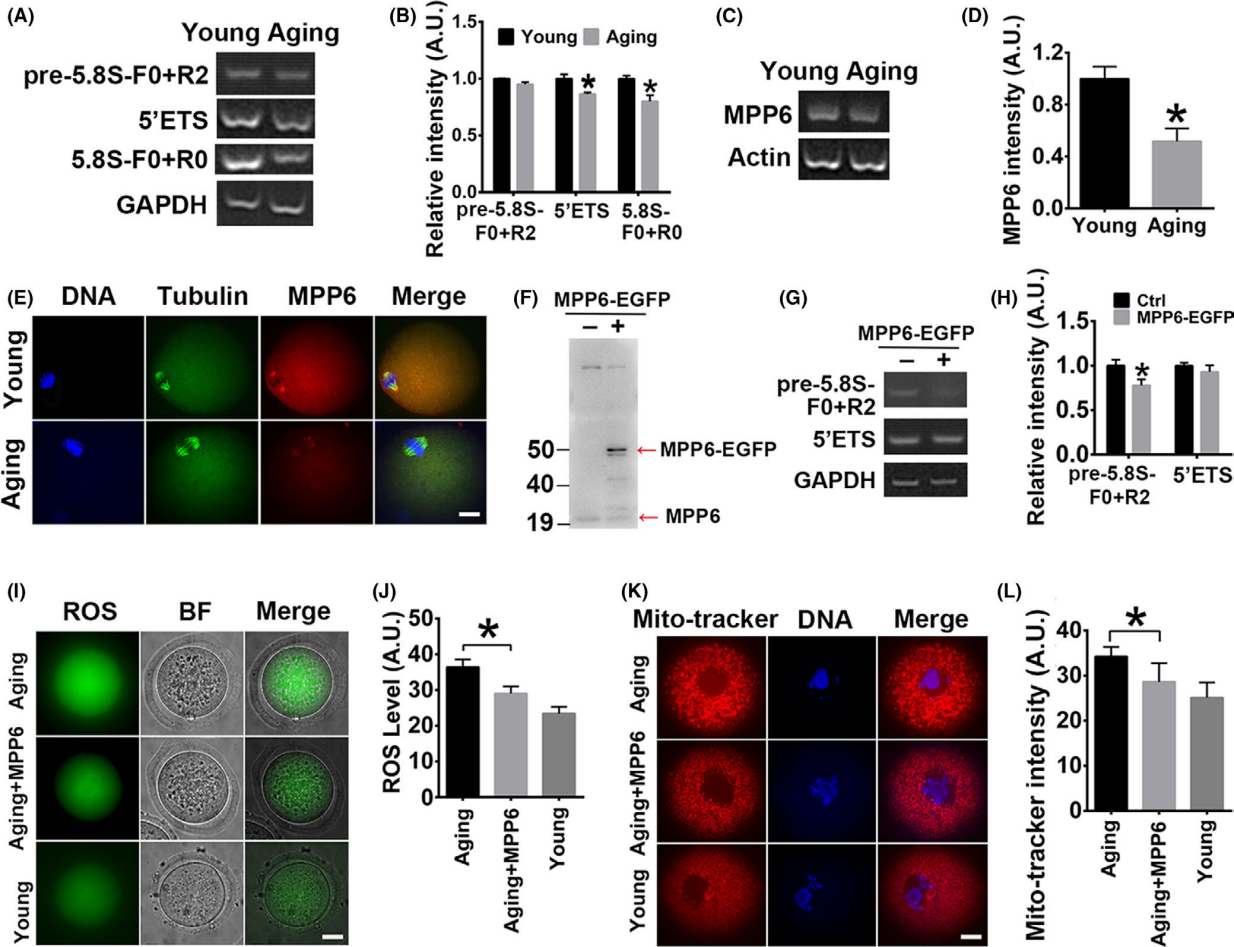
Ageing‐associated quality decrease of oocytes is partially correlated with reduced MPP6 level. (A) RT‐PCR showed that pre‐rRNA with 5'ETS primer pair and mature 5.8S rRNA with F0 + R0 primer pair both significantly decreased within ageing oocytes, and 5.8S pre‐RNA with F0 + R2 remained unchanged. (B) Quantification of (A). (C) RT‐PCR showed that the MPP6 mRNA level significantly decreased in ageing oocytes. (D) Quantification of (C). (E) Immunofluorescence staining showed that the MPP6 level significantly decreased within ageing oocytes. DNA in blue, tubulin in green, MPP6 in red. (F) Western blot showed that good overexpression of exogenous MPP6‐EGFP protein could be achieved by mRNA injection into oocytes. Red arrows marked endogenous MPP6 and exogenous MPP6‐EGFP. (G) RT‐PCR showed that MPP6‐EGFP overexpression significantly reduced 5.8S pre‐rRNA amplified with F0 + R2 primer pair, while did not change 5'ETS pre‐rRNA. (H) Quantification of (G). (I) ROS level in ageing oocytes was significantly higher than in young oocytes, while injection of MPP6‐EGFP mRNA into ageing oocytes significantly reduced the ROS level. (J) Quantification of (I). (K) Mitochondria aggregation in ageing oocytes was significantly higher than in young oocytes, while injection of MPP6‐EGFP mRNA into ageing oocytes significantly reduced the mitochondria aggregation. (L) Quantification of (K). Scale bar, 20 µm. *P < .05

Oocytes from 8‐month‐old retired mice, which corresponds to 35‐year‐old women, were used as ageing oocytes. Oocytes from 3‐week‐old mice were used as young oocytes. The PCR product with the 5.8S F0 + R0 primer pair inside the mature 5.8S rRNA, which will amplify both mature and pre‐5.8S rRNA, was significantly less in the ageing than young oocytes (Figure 8A,B, the intensity of gross 5.8S rRNA & pe‐rRNA amplified with F0 + R0 primer pair, young vs Ageing, 1.02 vs 0.82). The 5'ETS PCR product was also considerably reduced in ageing oocytes (Figure 9A,B, the intensity of pre‐rRNA amplified with 5'ETS primer pair, 0.98 vs 0.85). Accordingly, MPP6 mRNA level in aged mice was significantly reduced by almost 50% (Figure 9C,D, MPP6 intensity, 0.93 vs 0.48). Immunofluorescence also showed that the MPP6 protein signal within spindles was significantly decreased (Figure 9E). To further support the correlation between 5.8S pre‐rRNA level and MPP6 level, we injected in vitro‐transcribed MPP6‐EGFP mRNA into oocytes to successfully overexpress (OE) MPP6 protein (Figure 9F). RT‐PCR showed that the 5.8S pre‐rRNA amplified by F0 + R2 primer pair significantly reduced in MPP6‐OE oocytes compared with control oocytes (Figure 9G,H, the intensity of 5.8S pre‐rRNA amplified by F0 + R2, Ctr vs MPP6‐OE, 1.18 vs 0.95). These results, together with the upper MPP6‐DE results, suggested that MPP6 was required for the maturation of 5.8S rRNA.

Next, we examined how MPP6 overexpression would affect the quality of ageing oocytes. Results showed that the ROS level was significantly higher (Figure 9I,J), and mitochondria were significantly over‐aggregated in the ageing oocytes (Figure 9K,L), while MPP6 overexpression restored the ROS level and mitochondria intensity close to that in young oocytes (Figure 9I‐L). Together, these results suggested that decreased 5.8S rRNA maturation in the ageing oocytes might correlate with the decrease of MPP6.

## 4 DISCUSSION

MPP6 was previously screened but never verified as a female fertility factor. The results of the present study demonstrated, for the first time, that MPP6 is important for oocyte meiosis, quality and fertilization. And we also identified the pre‐rRNA region where MPP6 might bind and act. Moreover, we also found the correlations between oocyte ageing and the 5.8S rRNA level, oocyte ageing and the MPP6 level, MPP6 level and 5.8S rRNA maturation. Overall, these findings indicated that MPP6 might be an important female fertility factor.

FGOs store numerous maternal proteins to accomplish many cellular processes before fertilization. However, for many dynamic proteins, such as cyclin B1, active translation is still required for meiosis,^44^ probably to meet the increased level of cyclin B1 needed before MI or to replenish used "old" proteins.^45^ To ensure the active translation of maternal proteins, the ribosome must function normally; therefore, enzymatic rRNA should still exist in SN FGOs. Notably, several studies proposed that SN FGOs do not contain transcribed rRNA genes, pre‐rRNAs or pre‐ribosomes.^13^, ^14^, ^15^ However, these researchers only analysed the nuclear signal. A previous study showed that protein synthesis during MI of oocytes increased 3‐fold upon entry into meiosis 46 and, correspondingly, cytoplasm at MI stored more ribosomes.^47^ These findings are consistent with our FISH finding that SN oocytes have increased 5.8S rRNA in the cytoplasm.

Evidence that rRNA and pre‐rRNA exist in SN FGOs provided a basis for the function of MPP6 in SN FGOs because MPP6 is known to be a 5.8S rRNA processing factor. Our data showed that 5.8S pre‐rRNA, amplified specifically by F + R2, was significantly increased after MPP6 depletion. Subsequently, the localization of a checkpoint protein (BubR1), the activities of multiple meiotic kinases (MPF and Akt), and the levels of several indices of function (ROS, annexin V, mitochondria and ATP) all became abnormal. These changes may be due to damaged protein biogenesis of the ribosomes due to the reduction of 5.8S rRNA.

Finally, we found that the levels of both 5.8S pre‐rRNA and MPP6 were significantly decreased in oocytes from 8‐month‐old mice. Furthermore, supplementation with exogenous MPP6 could partially rescue oocyte quality, indicating that the reduction of rRNA and MPP6 might be at least partially responsible for the decreased oocyte quality in aged mice. A transcriptome study showed that 40 of 78 downregulated genes in the ovaries of ageing mice were ribosome‐related genes, such as Rps12, Rpl22l1, Rrs1 and Brix1.^43^ This supports the concept that ribosomal dysfunction is a major cause of the low quality of ageing oocytes.

Overall, our study, for the first time, shows that pre‐rRNA and rRNA exist in SN FGOs, and the pre‐rRNA maturation factor, MPP6, is important for the level of mature 5.8S rRNA and subsequent oocyte quality and meiosis. Therefore, MPP6 might be a potent marker for oocyte quality.

## CONFLICTS OF INTEREST

The authors declare that they have no conflicts of interest.

## AUTHOR CONTRIBUTIONS

Zhi‐Xia Yang, Zheng‐Rong Xia and Dong Zhang designed the research; Rui‐Rui Peng, Li‐Li Wang, Wen‐Yi Gao, Feng‐Yu Zhu, Fan Hu and Wen‐Tao Zeng performed most of the experiments, data collection and analysis. Li‐Ya Shi, Xi‐Chen Chen and Jing‐Yang Cai assisted in these processes. Rui‐Rui Peng prepared figures under the supervision of Zhi‐Xia Yang and Zheng‐Rong Xia. Dong Zhang wrote the manuscript with the assistance of Rui‐Rui Peng. Zhi‐Xia Yang and Zheng‐Rong Xia proofread and gave advice. All authors read and approved the final manuscript.

## Data Availability

The data that support the findings of this study are available from the corresponding author upon reasonable request.
