# Supplementary figures and images for: The 5.8S pre‐rRNA maturation factor, M‐phase phosphoprotein 6, is a female fertility factor required for oocyte quality and meiosis
Source: Cell Prolif. 2020 Jan 31;53(3):e12769. doi: 10.1111/cpr.12769 (PMC7106954; doi:10.1111/cpr.12769)

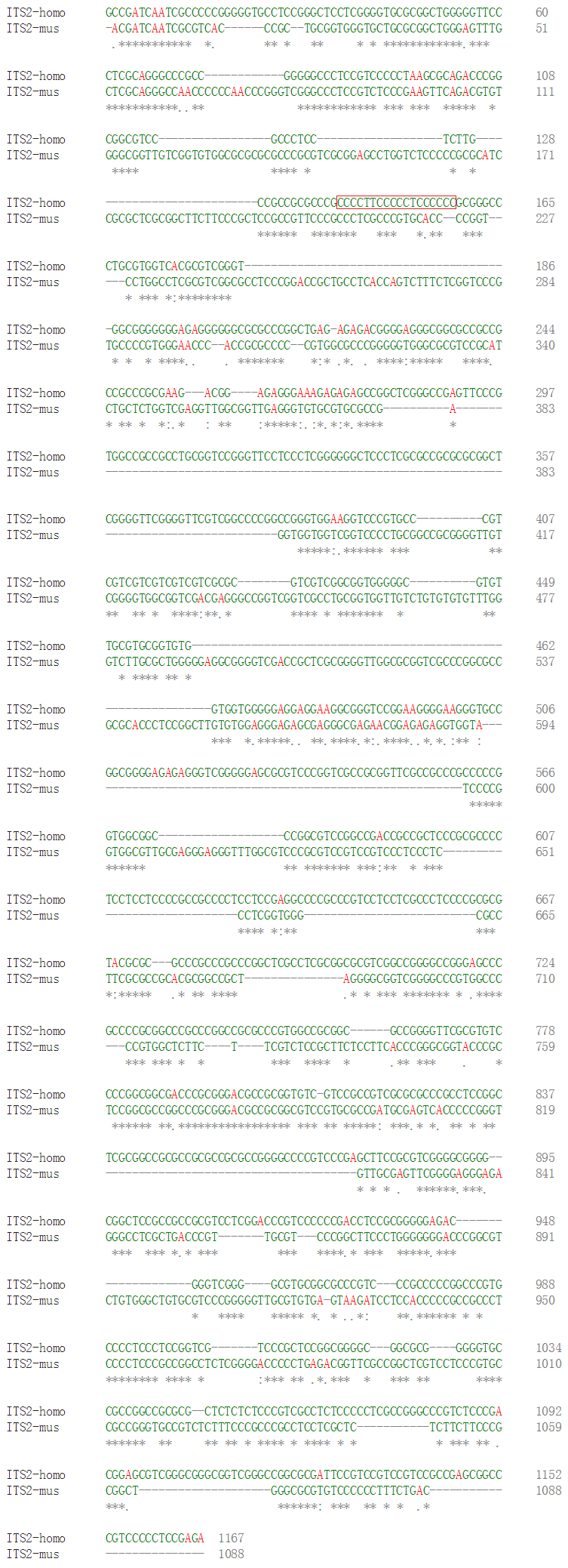

Supplement: Supplementary file 1 [file CPR-53-e12769-s001.tif]

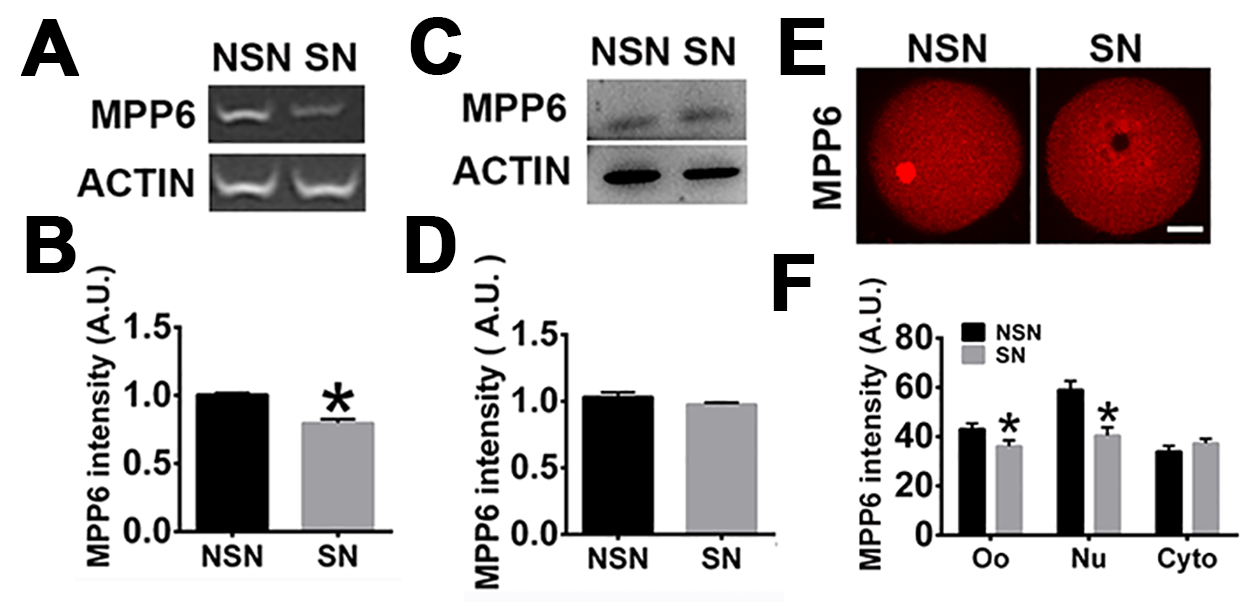

Supplement: Supplementary file 2 [file CPR-53-e12769-s002.tif]
