# Supplementary material for: The 5.8S pre‐rRNA maturation factor, M‐phase phosphoprotein 6, is a female fertility factor required for oocyte quality and meiosis
Source: Cell Prolif. 2020 Jan 31;53(3):e12769. doi: 10.1111/cpr.12769 (PMC7106954; doi:10.1111/cpr.12769)
Supplement: Supplementary file 3 [file CPR-53-e12769-s003.docx]

**Supplementary table 1**

| **Antibody Name** | **Company** | **Cat NO.** | **Dilution ratio** |
| --- | --- | --- | --- |
| anti-mphosph6 | Proteintech | 10695-1-AP | WB (1:500); IF (1:200); IHC (1:200) |
| anti-β-actin | Santa Cruz | A5316-100 | WB (1:1000) |
| anti-GAPDH | YEASEN | 30201ES60 | WB (1:5000) |
| anti-α-Tubulin | Santa Cruz | G3115 | IF (1:500) |
| anti-β-Tubulin | Santa Cruz | sc-5274 | IF (1:500) |
| anti-alpha tubulin (Acetyl Lys40) | Bioss | bsm-33235M | IF (1:500) |
| anti-BubR1 | Abcam, | ab28193 | IF (1:200) |
| anti-centromere CREST | Antibodies Incorporated | 15-234 | IF (1:500) |
| anti-cyclin B1 | Santa Cruz | B8566 | WB (1:500) |
| anti-Phosphorylation CDK1 (Thr14) | Aviva Systems Biology | OAAN02724 | WB (1:1000) |
| anti-Phosphorylation AKT (s473) | Cell Signaling Technology | 9271 | WB (1:1000) |
