# Supplementary material for: The 5.8S pre‐rRNA maturation factor, M‐phase phosphoprotein 6, is a female fertility factor required for oocyte quality and meiosis
Source: Cell Prolif. 2020 Jan 31;53(3):e12769. doi: 10.1111/cpr.12769 (PMC7106954; doi:10.1111/cpr.12769)
Supplement: Supplementary file 4 [file CPR-53-e12769-s004.doc]

**Supplementary table 2**

| **Name** | | **Sequence** | **Length** |
| --- | --- | --- | --- |
| GAPDH | Forward | 5'-ACCACAGTCCATGCCATCAC | 449bp |
|  | Reverse | 5'-CACCACCCTGTTGCTGTAGC |  |
| 5'ETS^1^ | Forward | 5'-GTCATTGTCCCGACGGTGTG | 188bp |
|  | Reverse | 5'-GAGGTAGACATCGACCGGAGC |  |
| ITS1^2^ | Forward | 5'-CAGACTCCATGACCCTCCTC | 242bp |
|  | Reverse | 5'-CAGGAACGAAACGAGACACG |  |
| F0+R0^3^ | Forward | 5'-GCTAGCTGCGAGAATTAATGTG | 110bp |
|  | Reverse | 5'-CAACCGACGCTCAGACAG |  |
| F0+R1^4^ | Forward | 5'- GCTAGCTGCGAGAATTAATGTG | 128bp |
|  | Reverse | 5'-GGTGACGCGATTGATCG |  |
| F0+R2^4^ | Forward | 5'- GCTAGCTGCGAGAATTAATGTG | 235bp |
|  | Reverse | 5'-ACCGACAACCGCCCACAC |  |
| F0+R3^4^ | Forward | 5'- GCTAGCTGCGAGAATTAATGTG | 312bp |
|  | Reverse | 5'-AACGGCGGAGCGGGAAGAAG |  |
| F0+R4^4^ | Forward | 5'- GCTAGCTGCGAGAATTAATGTG | 493bp |
|  | Reverse | 5'-ACCGCCACCCCGACAAC |  |
| F1+R5^4^ | Forward | 5'-ACACTTCGAACGCACTTGC | 700bp |
|  | Reverse | 5'-ACCGAGGGAGGGAGGGAC |  |
| F1+R6^4^ | Forward | 5'-ACACTTCGAACGCACTTGC | 796bp |
|  | Reverse | 5'-CGGAGACGAAGAAGAGCCAC |  |
| F1+R7^4^ | Forward | 5'-ACACTTCGAACGCACTTGC | 1097bp |
|  | Reverse | 5'-CGGGAAAGAGACGGCAC |  |
| TAMRA-5'ETS^5^ | | 5'- GAGGTAGACATCGACCGGAGCCCCCACCGCCAC | |
| FAM-R2^5^ | | 5'- ACCGACAACCGCCCACACGTCTGAACTTCGGGAG | |
